# Factors associated with adverse obstetric events following induction of labour: a retrospective study in a tertiary hospital in Ghana

**DOI:** 10.4314/ahs.v22i4.40

**Published:** 2022-12

**Authors:** Kwame Adu-Bonsaffoh, Joseph Seffah

**Affiliations:** 1 Department of Obstetrics and Gynaecology, University of Ghana Medical School, Accra, Ghana; 2 Department of Obstetrics and Gynaecology, Korle-Bu Teaching Hospital, Accra, Ghana

**Keywords:** Induction of labour, adverse outcomes, Ghana

## Abstract

**Background:**

Induction of labour (IOL) remains an indispensable intervention in obstetric practice; however, it may be associated with significant untoward perinatal outcomes. This study determined the major adverse outcomes of IOL and the associated factors at a tertiary hospital in Ghana

**Methods:**

Retrospective study involving women with singleton gestations, conducted at the Korle Teaching Hospital in Ghana. Multivariable logistic regression was used to explore the factors associated with adverse outcomes of IOL.

**Findings:**

A total of 195 women who had IOL were analysed with 161 (82.6%) and 34 (17.4%) undergoing vaginal and caesarean deliveries respectively. The main IOL methods used included Misoprostol (91.3%), Oxytocin (5.1%) and Foley's catheter (3.6%). Composite adverse perinatal outcomes occurred in 46 neonates (23.6%) comprising perinatal deaths (7.2%) and or NICU admission (21.0%). Caesarean delivery following IOL was significantly associated with nulliparity, gestational age <41 weeks, hypertensive disorders in pregnancy and birth weight ≥3.5kg. Gestational age <41 weeks and birth weight <2.5kg were significantly associated with adverse perinatal outcome. Five women (2.6%) had uterine rupture all of which occurred in the misoprostol group.

**Conclusion:**

Induction of labour may result in significant perinatal complications which are related to both maternal (nulliparity and hypertension) and fetal (gestational age and birth weight) factors. Strict selection criteria and continuous fetal-maternal monitoring are strongly recommended to improve the birth outcomes of IOL.

## Introduction

Induction of labour (IOL) describes the artificial stimulation of adequate uterine contractions (after fetal viability) prior to spontaneous or natural labour initiation with the prime objective of achieving vaginal childbirth.^1^–^3^ Labour induction is a common and very useful intervention in contemporary obstetrics constituting about 25% of childbirths at term in developed countries^1^, compared to approximately 4% and 12% in Africa and Asia respectively. 4 The main advantage of IOL resides in the facilitation of vaginal birth and avoidance of caesarean section (CS) with optimization of both maternal and neonatal outcomes in carefully selected expectant mothers.^1^,^2^,^5^

There are several globally recognizable clinical indications for IOL including prolonged pregnancy, intrauterine growth restriction and medical conditions in pregnancy hypertension and sickle cell disease.^2^,^3^,^6^ However, there is evidence that significant proportion of pregnant women undergo IOL without justifiable clinical indication with maternal request considered a major contributory factor.^7^ In such situations when a medically recognized indication cannot be readily identified the induction of labour is described as elective and is potentially associated with significant adverse outcomes.^7^,^8^

Generally, induction of may be is associated with significant maternal and perinatal risks. Therefore, the decision to prescribe the procedure to any pregnant woman must be based on sound clinical justification, and the expected benefits should outweigh the potential harms associated with the intervention.^1^,^2^,^5^,^8^–^10^ The main maternal complications of IOL include increased risk of caesarean section with its associated potential consequences such as increased blood loss, uterine rupture in subsequent maternities, longer hospital stays and increased cost. On the other hand, perinatal risks include birth asphyxia with poor APGAR scores, neonatal intensive care unit (NICU) admission and perinatal demise.^6^,^8^ Due to the potential harms associated with the procedure the World Health Organization (WHO) recommends that facilities should be available for adequate assessment of maternal and fetal conditions to prevent avoidable adverse outcomes. 1 Besides ensuring adequate intrapartum maternal and perinatal monitoring, identification of the factors associated with poor outcomes of induction of labour could predict and prevent major obstetric complications. In Ghana, there is limited research on induction of labour although it is frequently practiced in most health facilities. In a study carried out about 20 years ago in Ghana involving 160 women who underwent induction of labour, most (70%) were due to prolonged pregnancy followed by sickle cell disease (11.2%) and hypertensive disorders (9.4%). Induction with misoprostol resulted in vaginal delivery in 83% of the parturient with the rest undergoing caesarean section for various indications.^11^ Identification of the factors associated with poor induction of labour outcomes is underexplored in the country although such risk categorization may improve pregnancy outcomes. Integration of these associative factors of poor IOL outcomes in the clinical decision coupled with strict and careful selection criteria and appropriate intrapartum monitoring represents a viable primary approach to minimizing the associated adverse outcomes. This study determined the major adverse outcomes of IOL and the factors associated with adverse labour induction outcomes in tertiary hospital in Ghana.

## Methods

### Study design and site

This was a retrospective study conducted at the Korle Bu Teaching Hospital (KBTH), the biggest tertiary teaching hospital in Ghana conducting about 10,000 deliveries annually. The study included data on women who received maternity services between 1^st^ January to 30^th^ June 2015 at KBTH. This tertiary institution, situated in the capital, Accra, serves a population of over three million inhabitants. Most of the pregnant women delivering at the hospital are covered by National health insurance free delivery scheme. In KBTH, induction of labour is performed mainly using medical (misoprostol or oxytocin) and or surgical (amniotomy) after confirming the appropriateness of the indication for the procedure. Catheter induction is also undertaken in few indicated cases.

Prior cervical assessment using the modified Bishop score to determine the favourability of the cervix is mandatory. At the time when these patients were managed, induction of labour was mostly performed using vaginal misoprostol (50 microgram), inserted into the posterior vaginal fornix. The dose is usually repeated after 6 hours following appropriate maternal and fetal monitoring. If the maternal and fetal parameters are normal and there are no contraindications, subsequent doses of 50 microgram may be inserted up to a maximum of 4 doses. The procedure is considered failed if there are no uterine contractions after the maximum doses.

Following successful IOL with achievement of adequate uterine contractions and normal fetal-maternal parameters, progress of labour is monitored on a partograph based on the routine labour management protocol of the hospital. However, IOL cases receive extra vigilance in terms of fetal-maternal monitoring because of the increased risk of uterine hyper-stimulation with fetal heart rate abnormalities and uterine rupture.

### Eligibility criteria

The study included all women with singleton gestations who had induction of labour by various methods over the study period. We excluded women who had induction for termination of pregnancy prior to 28 weeks of gestation. Women with incomplete data on the final mode of childbirth and those with multiple gestations were also excluded.

In this study, induction of labour was defined as the artificial initiation of adequate uterine contractions in a pregnant woman who is not in labour with the aim of achieving vaginal birth.^2^,^5^ Failed induction of labour was defined as failure to achieve active labour after one cycle of treatment using a specific method of labour induction. 2,5,12,13

### Study size and bias

We included all the cases of induction of labour for the study period based on the inclusion and exclusion criteria. With the inclusion all the eligible women the risk of selection biased was minimized. Also, the list of potential participants obtained from the database was cross-checked with the admission and discharge entry register at the labour and recovery wards of the Maternity unit. This validation of the data was necessary to ensure that all the women who underwent induction of labour cases had been captured.

### Data sources and variables

The data collection process for this study occurred in two steps. Firstly, baseline data on all the women who had IOL over the study period were retrieved from the database of all the deliveries at the maternity unit of KBTH. The database comprises daily entry of the relevant clinical parameters of all women admitted at the Maternity unit of the hospital. The folder numbers of all the women who had undergone labour induction were then recorded after which study identification numbers were assigned to the women in the selected list.

The second part of the study consisted of retrieval of individual folders followed by collection and entry of the needed data into an excel spreadsheet. The specific data collected included maternal age in years, parity (number of previous births), gestational age at delivery (in weeks), mode of delivery (vaginal or caesarean), method of induction of labour (misoprostol, oxytocin and Foley's catheter), indications for induction and caesarean section, number of doses of misoprostol used, birth weight in kg, APGAR scores, sex of neonate, cervical assessment findings, number of antenatal visits and occurrence of hypertension in pregnancy among other parameters. The main maternal outcome was CS (mode of delivery) and the secondary outcomes include uterine rupture and maternal death. The main adverse perinatal outcome was the composite perinatal adverse outcome, defined as the occurrence of perinatal death or NICU admission.

### Data analysis

The data obtained was analysed using the SPSS version 20. Basic descriptive analysis was performed and the results were presented in percentages. Independent Student's T-test was used to compare the continuous variables between women who achieved vaginal delivery and those who had caesarean section following induction of labour. Univariate and multivariate logistic regression analyses were performed to determine the specific factors associated with caesarean section and composite adverse perinatal outcomes following induction of labour. P value of <0.05 was considered significant. For variables with missing data, complete case analysis was undertaken.

## Results

**Figure 1:**
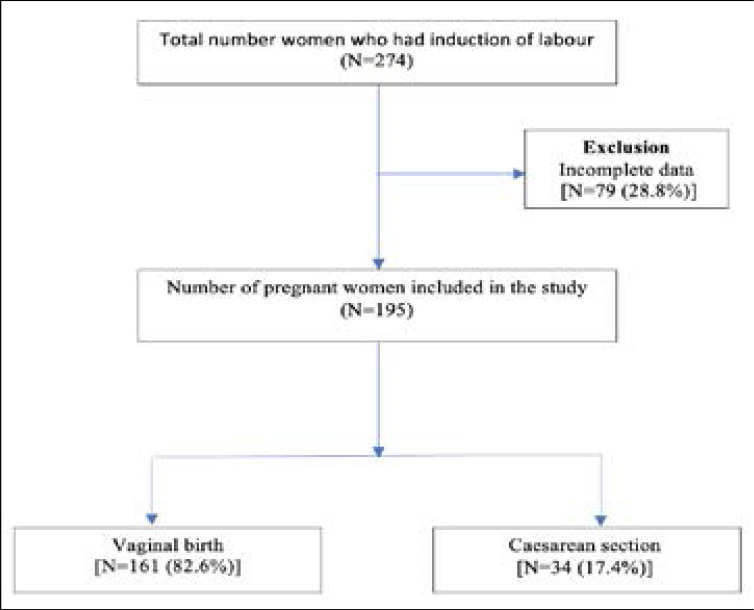
Flow chart indicating pregnant women who underwent induction of labour

Over the study period, there were 274 cases of induction of labour out of which 195 (71.2%) were included comprising 161 (82.6%) and 34 (17.4%) vaginal and caesarean deliveries respectively. Excluded from the final analysis were 79 (28.8%) cases of IOL on account of incomplete data on the final mode of childbirth and intrauterine deaths (Figure 1). The mean (±SD) maternal age for vaginal and caesarean groups were 29.24±5.88 and 28.72±6.39 years respectively. Prolonged pregnancy constituted the most common indication for IOL involving 136 (69.7%) women, followed by severe preeclampsia in 14.4% (n=28) , premature rupture of membranes in 5.1% (n=10) intrauterine fetal growth restriction in 4.6% (n=9) (4.6%), sickle cell disease in 2.6% (n=5) and other indications occurring in 3.6% (n=7). Regarding the gestational age, 10.8% (n=21) had IOL prior to term (<37 weeks). The obstetric and demographic characteristics of included women are shown in Table 1.

**Table 1:** Basic obstetric and demographic characteristics of women who had induction of labour

Variable	Vaginal delivery (N=161) n (%)	Cesarean section (N=34) n (%)
Maternal age		
< 35 years	129 (80.1)	27 (79.4)
≥ 35 years	32 (19.9%	7 (20.6)

Parity		
No previous birth (0)	50 (31.1)	20 (58.8)
Previous births (≥1)	111 (68.9)	14 (41.2)

Number of ANC		
Poor (<4)	13 (8.1)	2 (5.9)
Good (≥4)	148 (91.9)	32 (94.1)

Number of misoprostol*		
1 dose	29 (19.5)	3 (10.3)
2 doses	36 (24.2)	3 (10.3)
3 doses	33 (22.1)	8 (27.6)
4 doses	51 (34.2)	15 (51.7)

GA at Birth (weeks)^a^	39.68±3.20	39.44±2.77

GA at birth		
< 41 weeks	59 (36.6)	19 (55.9)
≥ 41 weeks	102 (63.4)	15 (44.1)
Birth weight (kg)^a^	2.55±0.95	2.65±1.22

Low birth weight		
No	113 (70.2)	24 (70.3)
Yes	48 (29.8)	10 (29.7)

* N=178; presented as mean±SD, ANC=Antenatal care; SD=standard deviation; Each dose of Misoprostol was 50 micrograms, GA=Gestational age ad delivery

The major methods used of induction of labour included the use of Misoprostol in 178 women (91.3%), oxytocin infusion in 10 women (5.1%) and Foley's catheter in 7 (3.6%) women. The use of membrane sweeping was not identified or documented in the medical records as a method of labour induction. Also, there was no documentation of amniotomy as a means of induction of labour. Regarding the mode of delivery 4 (57.1%), 8 (80.0%) and 149 (83.7%) women who underwent catheter, oxytocin and misoprostol inductions respectively delivered vaginally.

Overall, there was no significant difference between women who achieved vaginal delivery and those who had caesarean section following IOL with respect to maternal age, parity, antenatal visits, gestational age at delivery and birth weights (Table 1). However, there was a significant difference between women who achieved vaginal birth and those who failed to deliver vaginally regarding the number of doses of Misoprostol used for induction of labour.

The indications for CS among the women who could not achieve vaginal delivery following labour induction were fetal distress 12 (35.3%); cephalopelvic disproportion 10 (29.4%); failed induction 9 (26.5%); severe preeclampsia 2 (5.9%) and placental abruption 1 (2.9%). In the multivariate analysis, CS following IOL was significantly associated with nulliparity (adjusted OR 1.362, 95%CI 1.052–3.294), gestational age <41 weeks (adjusted OR 1.683, 95%CI 1.073–3.247), hypertensive disorders in pregnancy (adjusted OR 3.404, 95%CI 1.227–9.449) and birth weight ≥3.5kg (Adjusted OR 2.858, 95%CI 1.054–6.634) as shown in Table 2. There was no maternal mortality among the women who underwent induction of labour. Five women (2.6%) had uterine rupture and all these occurred in the misoprostol group.

**Table 2:** Factors associated with cesarean section following induction of labour at KBTH

Variable	Total N=195 n (%)	Emergency CS (N=32) n (%)	OR (95% CI)	P value	aOR* (95% CI)	P value
**Maternal age**						
<35 years ≥35 years	156 (80.0) 39 (20.0)	25 (16.0) 7 (17.9)	Reference 1.146 (0.455–2.885)	0.772	Reference 1.068 (0.263–1.840)	0.872
**Parity** No previous birth (0) Previous births (≥1)	70 (35.9) 125 (64.1)	18 (25.7) 14 (11.2)	2.745 (1.268–5.941) Reference	0.010	1.362 (1.052–3.294) Reference	0.041
**Gestational age at** **delivery** <41 weeks ≥41 weeks	78 (40.0) 117 (60.0)	19 (24.4) 13 (11.4)	2.576 (1.188–5.589) Reference	0.017	1.683 (1.073–3.247) Reference	0.039
**Number of** **antenatal visits** <4 ≤4	15 (7.7) 180 (92.3)	2 (13.3) 30 (16.7)	0.769 (0.165–3.586) Reference	0.738	1.169 (0.221–6.177) Reference	0.982
**Doses** **of Misoprostol**^m^ ≤2 ≥3	71 (39.9) 107 (60.1)	6 (8.5) 21 (19.6)	Reference 2.645 (0.010–6.928)	0.048	Reference 2.391 (0.903–6.331)	0.082
**Maternal** **hypertension** Yes No	36 (18.5) 159 (81.5)	12 (33.3) 20 (12.5)	3.475 (1.505–8.022) Reference	0.004	3.404 (1.227–9.449) Reference	0.019
**Birth weight** <3.5kg ≥3.5kg	173 (88.7) 22 (11.3)	24 (14.0) 8 (34.8)	Reference 3.289 (1.345–9.356)	0.010	Reference 2.858 (1.054–6.634)	0.040

* Adjusted for all variables in the table

m missing data=17; Each dose of Misoprostol was 50 micrograms

CS=Caesarean section

There was 14 perinatal deaths of which 12 (85.7%) were associated with induction of labour with misoprostol whereas 2 (14.3%) were associated with oxytocin. There were no perinatal deaths associated with catheter induction (Table 3). Poor Apgar score (less than 7) at five minutes occurred in 14 (7.2%) neonates following induction of labour. NICU admission occurred in 41 newborns (21.0%). Composite adverse perinatal outcome comprising perinatal death (n=14) or NICU admission (n=41) occurred in 46 newborns (23.6%). IOL prior to gestational age of 41 weeks (adjusted OR 2.318, 95%CI: 1.055–5.091) and birth weight <2.5kg (adjusted OR 2.243, 95%CI: 1.320–4.121) were significantly associated with composite adverse perinatal outcome (Table 4). Among the women who underwent IOL, 126 (64.6%) had their Bishop score (cervical assessment) documented out of which only 83 (65.9%) had complete documentation of the scoring system. The bishop score was therefore excluded from the multivariate analysis due to significant incomplete data

**Table 3:** Perinatal outcomes of induction of labour at Korle Bu Teaching Hospital

Induction method	Stillbirth (FSB) n (%)	Early neonatal death n (%)	Perinatal death n (%)
Catheter	0	0	0
Misoprostol	6 (94.1)	6 (85.7)	12 (91.7)
Oxytocin	1 (5.9	1 (14.3)	2 (8.3)
Total	7 (3.6) a	7 (3.7) b	14 (7.2)

a denominator=total births (n=195)

b denominator=total livebirths (n=188)

**Table 4:** Factors associated with poor (composite) perinatal outcome following induction of labour at KBTH

indicator	Poor perinatal outcome n (%)	OR (CI)	P value	aOR* (95%CI)	P value
**Maternal age** <35 years ≥35 years	36 (23.1) 10 (25.6)	Reference 1.149 (0.512–2.583)	0.736	Reference 1.032 (0.369–2.190)	0.952
**Parity** No previous birth (0) Previous births (≥1)	20 (28.6) 26 (20.8)	1.375 (0.830–2.274) Reference	0.222	1.021 (0.324–1.173) Reference	0.116
**Gestational age at** **delivery** <41 weeks ≥41 weeks	26 (33.3) 20 (17.1)	2.425 (1.237–4.755) Reference	0.010	2.318 (1.055–5.091) Reference	0.036
**Number of** **antenatal visits** <4 ≥4	4 (26.7) 42 (23.3)	1.195 (0.362–3.949) Reference	0.770	0.664 (0.169–2.449) Reference	0.538
**Doses** **of Misoprostol**^m^ ≤2 ≥3	16 (22.5) 26 (24.3)	Reference 1.103 (0.542–2.246)	0.786	Reference 0.883 (0.408–1.913)	0.753
**Maternal** **hypertension** Yes No	9 (25.0) 37 (23.3)	1.099 (0.475–2.544) Reference	0.825	0.753 (0.251–2.257) Reference	0.753
**Birth weight** <2.5kg ≥2.5kg	22 (37.9) 24 (17.5)	2.877 (1.444–5.734) Reference	0.003	2.243 (1.320–4.121) Reference	0.007

* Adjusted for all variables in the table

m missing data 4

aOR=adjusted Odds Ratio; CI=confidence interval

## Discussion

In this study, the outcomes of induction of labour and important factors associated with adverse outcomes have been highlighted. The study included 195 induction of labour cases out of which 82.6% achieved vaginal birth whiles 17.4% underwent caesarean section for various reasons. Composite adverse perinatal outcome comprising perinatal death and or NICU admission occurred in 23.6%. Factors associated with caesarean delivery were nulliparity, gestational age <41 weeks, hypertensive disorders and birth weight ≥3.5kg whiles gestational age <41 weeks and birth weight <2.5kg were significantly associated with adverse perinatal outcome.

The caesarean rate determined in our study is consistent with the 18% reported by Khan et al in Pakistan.^14^ More recently, a similar study by Lawani et al determined CS rate of 24.3% following IOL in a tertiary hospital in Nigeria. 15 In this study, prolonged pregnancy constituted the most common indication for induction of labour accounting for 69.7%, higher than the 45.8% determined in Nigeria.^15^

The main objective of IOL is to achieve safe vaginal delivery, and caesarean section performed after initiation of the induction process is considered an adverse outcome. In this study, caesarean delivery following IOL was significantly associated with nulliparity, gestational age <41 weeks, hypertensive disorders and birth weight ≥3.5kg. The significant association between nulliparity and CS following IOL is consistent with a recent study by Khan et al.^14^ Maternal age greater 35 years was not significantly associated with caesarean section, contrary to the report by Jonsson et al.^16^ The use of three or more doses of 50 microgram of misoprostol showed a potential association with emergency CS which might be partly attributed to uterine hyper-stimulation. Similarly, hypertensive disorders in pregnancy were also associated with increased frequency of caesarean birth. This might be attributable to uncontrollable blood pressures as well as fetal distress associated with the use of some antihypertensives such as hydralazine.

Regarding those who failed to achieve vaginal birth following labour induction, fetal distress was the leading indication for CS, responsible for 35.3%. Fetal distress in association with labour induction is usually due to uterine hyper-stimulation, defined as five or more uterine contractions within a period of ten minutes with fetal heart abnormalities. Hyper-stimulation reduces utero-placental flow resulting in placental hypoperfusion and fetal hypoxia. In Nigeria, Lawani et al also determined that fetal distress was the commonest indication for caesarean section following induction of labour with misoprostol.^15^

Similarly, failed IOL constituted about 27% of women who had CS. In this study, failed IOL was defined as failure to achieve active labour following the use of an appropriate method for labour induction.^2^,^5^,^12^,^13^ In another parlance, failed induction is defined as failure to achieve vaginal delivery after the induction process irrespective of the reason for caesarean delivery.^13^ It is important to acknowledge that failed induction of labour due to lack of uterine contractions does not inevitably constitute a justifiable indication for caesarean section.^1^,^2^,^5^ In such situations, adequate client education with case-specific counselling is indispensable in deciding on further management (trial of IOL again or caesarean section). The preference of the woman and clinical circumstances are paramount in the final decision.^5^

Concerning perinatal outcome indicators, IOL prior 41 weeks and birth weight <2.5kg were significantly associated with composite adverse perinatal outcome, defined as the occurrence of perinatal death and or NICU admission. Most of the adverse perinatal outcomes were associated with the use of misoprostol and less commonly with oxytocin. There was no perinatal mortality among the women who had catheter induction of labour. More recently, Kruit et al determined that IOL using Foley catheter among women with prolonged pregnancy was not associated with increased perinatal morbidity compared with spontaneous onset of labour but resulted in a considerable increase in CS rate among nulliparous women. 17 In our study, Foley's catheter was used for induction of labour in 3.6% of the women induced and there were no perinatal adverse outcomes.

The most common method of IOL in this study was the use of vaginal misoprostol which accounted for 91% which is lower than the 78.2% determined by Lawani et al in Nigeria.^15^ Induction of labour with oxytocin was found to be the commonest induction method in a study in Ethiopia^18^. In KBTH, the protocol for induction of labour using misoprostol has evolved over the past decades with specific revisions aimed at improving both the perinatal and maternal outcomes. Over past decades, the traditional protocol for IOL with misoprostol in the hospital was 50 microgram 4 hourly for four doses. However, the protocol was revised to 50 microgram 6 hourly for four doses over a decade ago. The need for the change in protocol was necessitated by the high frequency of untoward obstetric outcomes such as uterine rupture and perinatal losses. It is well acknowledged that 25 microgram misoprostol is the recommended vaginal dose for IOL by WHO.^1^ There exists a real clinical challenge in obtaining this 25microgram fragments accurately as the currently available formulation in the country is 200 microgram tablet which is usually divided into four. The WHO recommends the use of low-dose vaginal misoprostol (25 microgram, 6-hourly) for IOL to reduce both maternal and perinatal outcomes.^1^ In low resource settings where stringent monitoring of maternal and fetal parameters is not adequately assured the use of the 25 micrograms of misoprostol formulations will significantly improve IOL outcomes. Moving forward, we recommend that misoprostol in 25 microgram formulations be made freely available in low resource countries to avert the situational challenges associated with dividing the 200 microgram tablets into 50 or 25 micrograms.

More recently, the use of oral misoprostol solution (OMS) for IOL is increasing globally and has been proven to be equally effective. In a recent randomized trial in China, titrated OMS for IOL resulted in a lower CS rate (21.7% versus 27%) and tachysystole with fetal heart rate abnormalities (3.6% versus 8.6%) compared to vaginal dinoprostone.^19^ The use of OMS has been found to be equally effective compared with vaginal route in systematic review by Hofmyer et al.^20^ Similarly, OMS was found to be associated with lower induction to delivery interval and less side effect compared to vaginal misoprostol in a recent RCT in Egypt. In Ghana, the use of OMS is not well integrated.^21^ Given the improved safety profile and efficacy, we recommend careful introduction and integration OMS for IOL in the country to obviate the challenges associated obtaining 25 microgram formulations of misoprostol.

There is evidence that membrane sweeping at term decreases the necessity for undertaking formal IOL for prolonged pregnancy as it increases the likelihood of spontaneous onset of labour.^1^ In our study, none of the women with prolonged pregnancy had sweeping of membranes done based on the medical records. This finding could be partly due to non-routine practice of membrane sweeping routinely for women with prolonged pregnancy or poor clinical documentation. In our opinion, membrane sweeping should be offered routinely to pregnant women after 40 weeks (and documented) to reduce the incidence of prolonged pregnancy and the need for formal labour induction. Cervical massage in the vaginal fornices is recommended when the cervix is closed in which case sweeping of membranes is contraindicated. In such situations, cervical massage may achieve similar effects as membrane sweeping.^5^

## Limitations and strengths

In this study, Bishop score based on cervical assessment prior to IOL was not included in the analysis because the component parameters were not adequately documented in the medical records in more than half of the women. Significant proportion of women whose Bishop scores were reported had incomplete documentation of the various components of the scoring system.

## Conclusion

Induction of labour remains an essential obstetric intervention although the induction process has the potential for adverse obstetric sequelae including caesarean section and perinatal deaths. Caesarean delivery following induction of labour was significantly associated with nulliparity, gestational age <41 weeks, hypertensive disorders and birth weight >3.5kg. Composite adverse perinatal outcome of IOL is associated with gestational age <41 weeks and birth weight <2.5kg. Strict selection criteria and continuous monitoring are strongly recommended to improve the pregnancy outcomes of induction of labour.

## Abbreviations

CS: Caesarean section
IOL: Induction of labour
KBTH: Korle Bu Teaching Hospital
NICU: Neonatal Intensive Care Unit
WHO: World Health Organization
